# Supplementary material for: Development of an rpS6-Based Ex Vivo Assay for the Analysis of Neuronal Activity in Mouse and Human Olfactory Systems
Source: Int J Mol Sci. 2024 Dec 7;25(23):13173. doi: 10.3390/ijms252313173 (PMC11642283; doi:10.3390/ijms252313173)
Supplement: Supplementary file 1 [file ijms-25-13173-s001.zip › ijms-3299879-supplementary.pdf]

Supplementary Files for

Development of an rpS6-Based Ex Vivo Assay for the Analysis of Neuronal Activity in Mouse and Human Olfactory Systems.

Emma Broillet-Olivier, Yaëlle Wenger, Noah Gilliand, Hugues Cadas, Sara Sabatasso, Marie-Christine Broillet and Julien Brechbühl

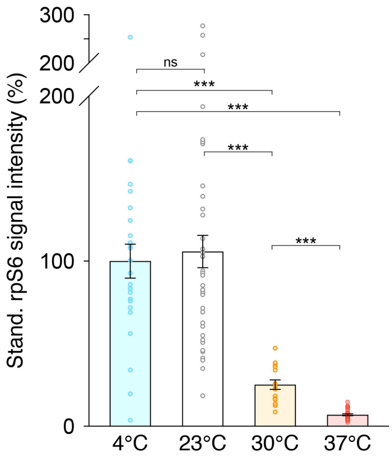

Figure S1. Statistical analysis of rpS6 temperature-dependent signal intensity in OMP-GFP mice. Assessment of the temperature effect on the rpS6 signal in the GG of homozygous OMP-GFP mice at different temperatures (4°C, 23°C, 30°C, and 37°C). Data are expressed as a standardized percentage of rpS6 signal intensity and represented as mean ± SEM with aligned dot plots for a minimum of 3 GG sections per animal, from at least 2 mice per condition. Comparisons between conditions were performed using two-tailed Welch’s t-tests or Mann-Whitney U-tests, *** $p < 0.001$, and ns for non-significant.

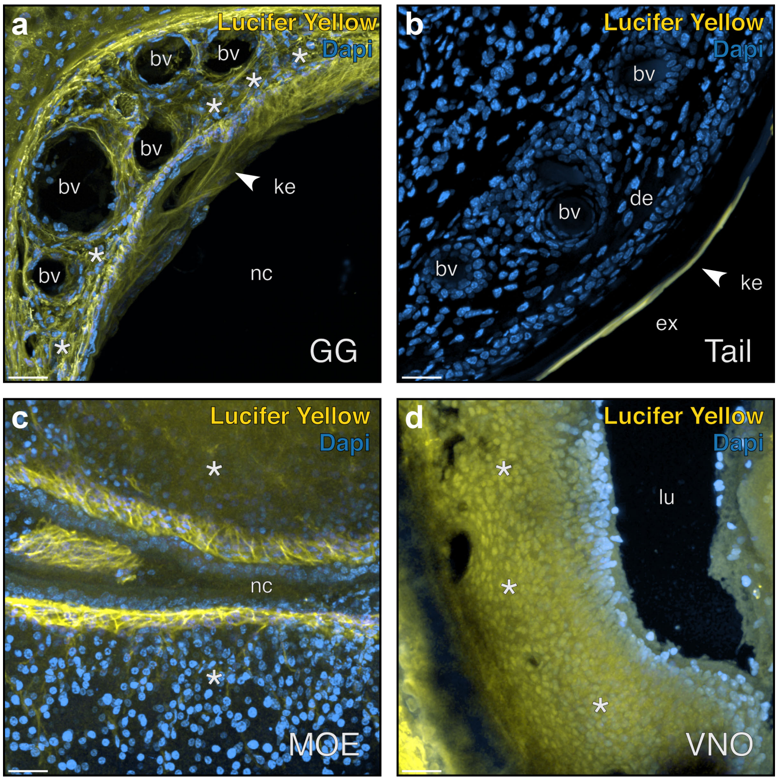

Figure S2. Assessment of odorant accessibility. Lucifer yellow was used as a hydrophilic dye (in yellow) to mimic the accessibility of hydrosoluble odorant molecules in our rpS6-based *ex-vivo* assay. Staining was evaluated in the GG (**a**), serving as a permeable tissue control; in the tail (**b**), used as an impermeable tissue control; and in the MOE (**c**) and VNO (**d**). In contrast to the olfactory subsystems (**a**, **c**, and **d**), the absence of Lucifer yellow staining in the dermal layer (de; **b**) of the tail's skin confirms its impermeability to hydrosoluble molecules. Key anatomical features are annotated: nasal cavities (nc; in **a**, **c**), lumen (lu; in **d**), keratinized epithelia (ke; in **a**, **b**), blood vessels (bv; in **a**, **b**), the external environment (ex; in **b**), and sensory epithelia (white asterisks; in **a**, **c**, **d**). The merged views include DAPI nuclear counterstaining (blue; **a**-**d**). Observations were performed on at least 5 cryosections per tissue obtained from 4 OMP-GFP mice. Scale bars: 30 µm (**a**-**d**).

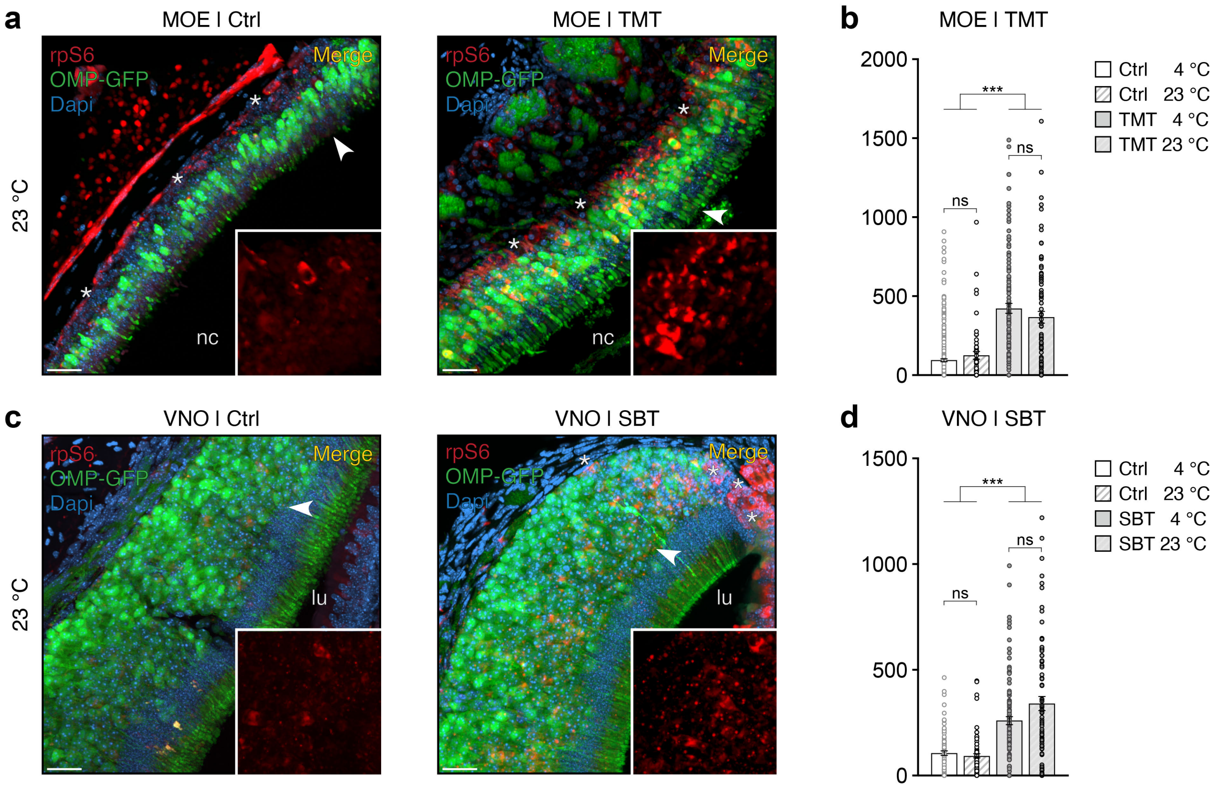

Figure S3. Analysis of rpS6-based odorant signals in the MOE and VNO at lower conditioning temperatures. (**a**) Representative immunostaining of the rpS6 signal (red) in the MOE of OMP-GFP mice (OMP-GFP signal in green) under non-stimulated control conditions (Ctrl, left panel) and after TMT stimulation (TMT, right panel), shown here at a conditioning temperature of 23°C. (**b**) Statistical analysis of the effects of TMT stimulation on GFP-positive OSNs in the MOE. Data are presented for conditioning temperatures of 4°C and 23°C. (**c**) Representative immunostaining of the rpS6 signal (red) in the VNO of OMP-GFP mice under non-stimulated control conditions (Ctrl, left panel) and after SBT stimulation (SBT, right panel) at 23°C. (**d**) Statistical analysis of the effects of SBT stimulation on GFP-positive OSNs in the VNO. Data are presented for conditioning temperatures of 4°C and 23°C. White asterisks indicate potential proliferative basal GFP-negative cells expressing the rpS6 signal, located above the basal lamina in the MOE (**a**) or within marginal compartments in the VNO (**c**). Nasal cavities (nc) in the MOE (**a**) and lumens (lu) in the VNO (**c**) are labeled. White arrowheads denote regions with magnified views of the rpS6 signal (**a**, **c**). Scale bars: 20 µm (**a**, **c**). Merged images include nuclear DAPI staining (blue, **a**, **c**). Stimulations under control and odorant conditions are represented by solid white and gray bars for 4°C and dashed white and gray bars for 23°C, respectively. (**b**, **d**). A minimum of 4 tissue sections per animal from at least 3 mice per condition were analyzed. Statistical comparisons between conditions were performed using two-tailed Welch's *t*-tests or Mann–Whitney *U*-tests, ***$p < 0.001$, and ns for non-significant.
